# Novel Insertion/Deletion Polymorphisms and Genetic Studies of the Shadow of Prion Protein (*SPRN*) in Raccoon Dogs

**DOI:** 10.3390/ani14243716

**Published:** 2024-12-23

**Authors:** Da-In Choi, Mohammed Zayed, Eun-Jee Na, Jae-Ku Oem, Byung-Hoon Jeong

**Affiliations:** 1 Korea Zoonosis Research Institute, Jeonbuk National University, Iksan 54531, Republic of Korea; cdi68@jbnu.ac.kr (D.-I.C.); mzayed2@vet.svu.edu.eg (M.Z.); 2 Department of Bioactive Material Sciences, Institute for Molecular Biology and Genetics, Jeonbuk National University, Jeonju 54896, Republic of Korea; 3 Department of Surgery, College of Veterinary Medicine, South Valley University, Qena 83523, Egypt; 4 Laboratory of Veterinary Infectious Diseases, College of Veterinary Medicine, Jeonbuk National University, Iksan 54596, Republic of Korea; ejna1212@naver.com (E.-J.N.); jku0623@jbnu.ac.kr (J.-K.O.)

**Keywords:** prion, *SPRN*, Sho, insertion/deletion variants, SNP, raccoon dog

## Abstract

Prion diseases, or transmissible spongiform encephalopathies (TSEs), are a group of invariably fatal neurodegenerative disorders. One of the candidate genes involved in prion diseases is the shadow of the prion protein (*SPRN*) gene. Raccoon dogs, a canid, are considered to be a prion disease-resistant species. To date, the genetic polymorphisms of the *SPRN* gene and the predicted protein structure of the shadow of prion protein (Sho) have not been explored in raccoon dogs. *SPRN* was amplified using polymerase chain reaction (PCR). We also investigated the genetic polymorphisms of *SPRN* by analyzing the frequencies of genotypes, alleles, and haplotypes, as well as the linkage disequilibrium among the identified genetic variations. In addition, *in silico* analysis with MutPred-Indel was performed to predict the pathogenicity of insertion/deletion polymorphisms. Predicted 3D structures were analyzed by the Alphafold2. We found a total of two novel synonymous single nucleotide polymorphisms and three insertion/deletion polymorphisms. In addition, the 3D structure of the Sho protein in raccoon dogs was predicted to resemble that of the Sho protein in dogs. This is the first study regarding the genetic and structural characteristics of the raccoon dog *SPRN* gene.

## Simple Summary

Prion diseases are rare and fatal neurodegenerative disorders associated with the shadow of the prion protein (*SPRN*) gene. Raccoon dogs, members of the Canidae family, may exhibit resistance to these diseases, yet the specific factors underlying this resistance remain unidentified. In this study, we amplified and analyzed the *SPRN* gene in raccoon dogs using PCR and DNA sequencing, identifying five novel genetic polymorphisms. *In silico* analysis assessed the pathogenic potential of these insertion/deletion polymorphisms, indicating that they are non-pathogenic. This research marks the first exploration of the genetic and structural characteristics of the *SPRN* gene in raccoon dogs.

## 1. Introduction

Prion diseases are a group of degenerative brain diseases that affect humans and other mammals [1]. An increasing number of animals, including cattle, sheep, goats, and deer, can be infected with prion diseases [2,3]. A conformational change from the normal prion protein (PrP^C^) to its abnormal form (PrP^Sc^) induces prion diseases. The PrP^C^ plays a role in multiple physiological activities, such as cell growth, adhesion, differentiation, and neurogenesis [4]. In prion disease, the deposition of PrP^Sc^ aggregates in the brain is thought to contribute to the neuropathological features of the disease and lead to neurological damage, resulting in severe neurological symptoms and, eventually, death [5].

Many factors affect susceptibility to prion disease, including the prion protein (*PRNP*) gene [6]. A study was conducted to investigate the association between genetic variations in the *PRNP* gene and the susceptibility of sika deer to chronic wasting disease (CWD) [7]. However, one of the factors is the possibility of additional candidate genes influencing prion disease susceptibility rather than just the *PRNP* gene. Considered to be involved in prion pathogenesis, the shadow of prion protein (*SPRN*) gene is an intriguing candidate gene [8]. Human studies have previously found an association between variant and sporadic Creutzfeldt–Jakob disease (CJD) incidence and mutations in the *SPRN* gene [9]. The shadow of prion protein (Sho), which is expressed by the *SPRN* gene, has a neuroprotective effect and is found mainly in the neurons of the central nervous system (CNS) [10]. The Sho protein exhibits strong similarity to the prion protein and is a glycosylphosphatidylinositol (GPI) anchor protein [10,11]. It has been reported that *SPRN* and *PRNP* have diverged from a common ancestral gene into genes that may have acquired new biological activities in addition to some shared functions [12]. The homologs of disease-related genes are anticipated to enhance the understanding of physiological and pathogenic mechanisms and could be considered potential targets for drug development [13]. Previous studies based on cell culture models showed that the rate of PrP^Sc^ conversion rose in a dose-dependent fashion with the concentration of the Sho, suggesting that Sho affects the folding pathway of prion proteins [14].

The raccoon dog (*Nyctereutes procyonoides*), a canid, is distributed across southeastern Siberia, Korea, China, Japan, Vietnam, and East Europe [15]. During the bovine spongiform encephalopathy (BSE) outbreak, no documented cases of prion infections were observed in dogs. A protein misfolding cyclic amplification assay revealed that dog PrP was found to be resistant to conversion by various prion agents, including BSE, scrapie, and CWD [16]. Fernández-Borges et al. examined the canine PrP amino acid sequence and performed *in silico* structural analysis, identifying a key amino acid linked to its resistance to prion disease. A transgenic mouse model study further suggested that Asp163 in dogs plays a critical role in this prion resistance [17]. Raccoon dogs act as carriers and reservoirs for many transmissible diseases, including vector-borne pathogens and zoonoses, and thus represent a potential hazard to humans and other animals’ health, as well as to biological diversity and ecological initiatives [18]. Their high population growth has raised concerns regarding the disruption of the ecosystem and its role in the spread of zoonotic diseases [19]. Currently, there is a shortage of research on objective strategies used to assess the integrity and capability of the neural system in the raccoon dog. Additionally, the susceptibility of raccoon dogs to prion infections has not been explored to date. Furthermore, while polymorphisms in the *PRNP* gene have been documented [20], no studies have investigated *SPRN* polymorphisms in this species.

To explain the potential for the prion resistance in raccoon dogs, we utilized the polymerase chain reaction (PCR) to amplify *SPRN* gene sequence and conducted amplicon sequencing to detect genetic variations. In addition, we examined the genotype, allele, linkage disequilibrium (LD), and haplotype frequencies of single nucleotide polymorphisms (SNPs) of the raccoon dog *SPRN* gene. We also analyzed LD between SNPs in the raccoon dog *SPRN* and *PRNP* genes. Furthermore, we evaluated the effects of insertion/deletion polymorphisms of the raccoon dog *SPRN* gene using an *in silico* tool. Finally, we attempted to predict the 3D structure of Sho in the raccoon dog using AlphaFold2.

## 2. Materials and Methods

### 2.1. Ethical Statement

The National Institute of Environmental Research in the Republic of Korea and the College of Veterinary Medicine at Jeonbuk National University donated tissue samples from raccoon dogs; these samples had been stored in a deep freezer (−80 °C). All experimental guidelines were approved by the Institutional Animal Care and Use Committee (IACUC) of Jeonbuk National University (CBNU 2020-083).

### 2.2. Genomic DNA

Genomic DNA was isolated from 20 mg of raccoon dog tissue samples using a Bead Genomic DNA Prep Kit (BioFACT, Daejeon, Republic of Korea).

### 2.3. Genetic Analysis of the Raccoon Dog SPRN Gene

To amplify the raccoon dog *SPRN* gene, a PCR was performed with primers, including *SPRN*-forward (GTCCCCGAGCCCCTGACC) and *SPRN*-reverse (CCAGGTCGGTGCAGGAGG). These primers were designed with reference to the raccoon dog *SPRN* gene (Gene ID: 129523253). The 25 µL PCR mixture contained 1 µL of each 10 mM dNTP mix, 5 µL of 5× band helper, 2.5 µL of 10× H-star Taq reaction buffer, 1 µL of each primer (10 µM), and 0.2 µL of H-star Taq DNA polymerase (BIOFACT, Daejeon, Republic of Korea). The PCR was carried out under the following conditions: 98 °C for 15 min for the denaturation step, 35 cycles of 98 °C for 20 s, 58 °C for 30 s, and 72 °C for 1 min for annealing and extension steps and 1 cycle of 72 °C for 5 min for the final extension step. Purification steps were performed using a FavorPrep gel/PCR Purification Mini Kit (FAVORGEN, Pingtung County, Taiwan). Data from a previous study were used to compare the linkage between SNPs in the *SPRN* and *PRNP* genes of raccoon dogs [21]. Briefly, PCR was conducted to amplify the raccoon dog *PRNP* gene using gene-specific primers, including *PRNP*-forward (GAGCACACGTAGGATGCTGA) and *PRNP*-reverse (CCTCCCCCAACCTGTAAAA). These primers were designed with reference to the raccoon dog *PRNP* gene (Gene ID: EU341507.1).

### 2.4. Genetic Variations of the SPRN Gene Across Different Species

Data on *SPRN* gene polymorphisms were gathered from previously published studies. Subsequently, we used Microsoft PowerPoint version 2407 to compare and present these polymorphisms.

### 2.5. Multiple Sequence Alignments

Amino acid sequences of Sho were retrieved from GenBank at the National Center for Biotechnology Information (NCBI), including those of humans (*Homo sapiens*, NP_001012526.2), cattle (*Bos taurus*, NP_001073790.1), sheep (*Ovis aries*, NP_001156033.1), goats (*Capra hircus*, XP_017896762.1), red deer (*Cervus elaphus*, XP_043781154.1), horses (*Equus caballus*, XP_023492126.1), rabbits (*Oryctolagus cuniculus*, XP_008268877.2), dogs (*Canis lupus familiaris*, XP_038435137.1), and raccoon dogs (*Nyctereutes procyonoides*, XP_055201840.1). The amino acid sequences of Sho were aligned with ClustalW, employing progressive alignment techniques.

### 2.6. MutPred-Indel

An *in silico* tool was utilized to predict the impact of one amino acid substitution on the protein function. MutPred-Indel (http://mutpred2.mutdb.org/mutpredindel/ (accessed on 20 March 2024)) is a program that distinguishes between functional residue types affected by non-frameshifting insertion/deletion variation and predicts pathogenicity. For each variant, MutPred-Indel predicted a pathogenicity score ranging from zero to one, where variants with scores close to one have a higher likelihood of being pathogenic [21].

### 2.7. AlphaFold2

The 3D structure of Sho was predicted using AlphaFold2, which is based on machine learning techniques (https://colab.research.google.com/github/sokrypton/ColabFold/blob/main/AlphaFold2.ipynb, accessed on 20 March 2024). Confidence in the modeling was evaluated using the predicted local distance difference test (pLDDT) score, ranging between 0 and 100. Higher pLDDT scores signify greater confidence in the accuracy of the residue structure, while lower scores can suggest that the residues are located in intrinsically disordered regions of the protein.

### 2.8. Statistical Analyses

The analyses of LD and haplotypes were carried out using Haploview version 4.2 (Broad Institute, Cambridge, MA, USA) [22]. To assess Hardy–Weinberg equilibrium (HWE), the chi-square test was utilized [23].

## 3. Results

### 3.1. Identification of Novel Polymorphisms in Raccoon Dog SPRN Gene

**Figure 1:**
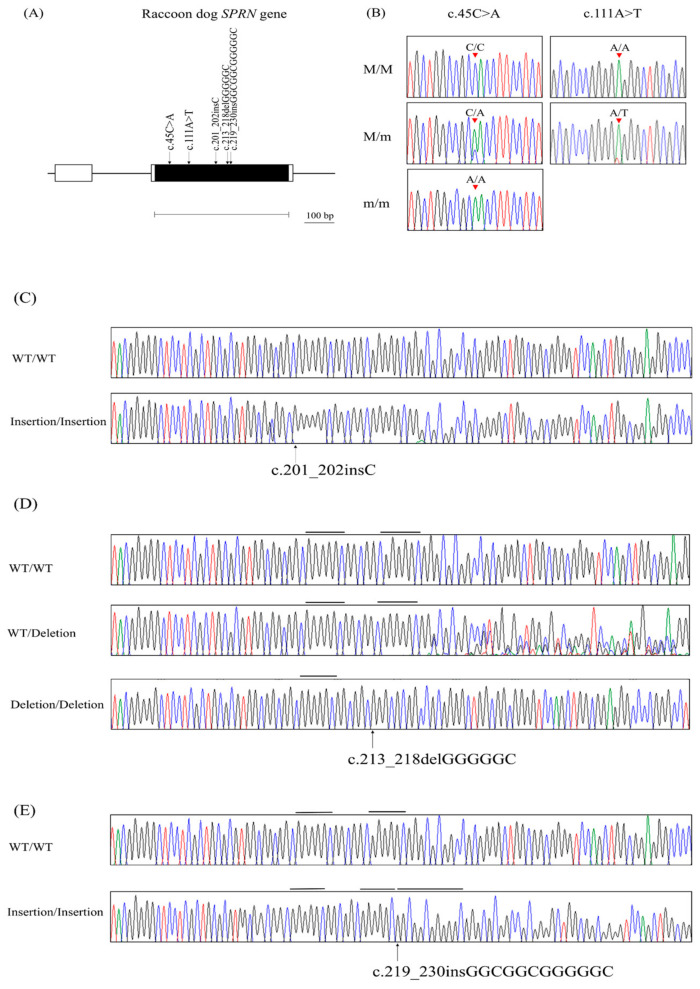
Identification of single-nucleotide polymorphisms (SNPs) in the raccoon dog of the shadow of prion protein gene (*SPRN*). (**A**) Gene map and polymorphisms identified in the raccoon dog *SPRN* gene. Shaded block represents the open reading frame (ORF) within the exon, with arrows pointing to the novel polymorphisms identified in this study. The outlined horizontal bar denotes the sequenced region. (**B**) Electropherograms show two novel synonymous SNPs, with red arrows indicating their locations. M/M represents major homozygotes, M/m represents heterozygotes, and m/m represents minor homozygotes. (**C**) Electropherogram of c.201_202insC polymorphism, indicated by a black arrow. (**D**) Electropherogram of c.213_218delGGGGGC polymorphism, indicated by a black arrow. (**E**) Electropherogram of c.219_230insGGCGGCGGGGGC polymorphism, indicated by a black arrow. WT represents wild-type allele. Insertion represents insertion allele. Deletion represents deletion allele. Colored peaks represent each base of the DNA sequence (green for adenine; red for thymine; blue for cytosine; black for guanine).

To identify the genetic polymorphisms of the raccoon dog *SPRN* gene, we performed DNA sequencing in 64 raccoon dogs. In total, we found two novel SNPs, including c.45C > A and c.111A > T, as well as three novel insertion/deletion polymorphisms, including c.201_202insC, c.213_218delGGGGGC, and c.219_230insGGCGGCGGGGGC in the open reading frame (ORF) region (Figure 1 and Table 1). In addition, we investigated the extent of LD among the *SPRN* polymorphisms using r^2^ values (Table 2). Strong LD (r^2^ > 0.333) was not observed. We also investigated LD between raccoon dog *SPRN* and *PRNP* polymorphisms using r^2^ values. In Table 3, strong LD (r^2^ > 0.333) was not observed between raccoon dog *PRNP* and *SPRN* polymorphisms. Furthermore, we performed haplotype analysis on the SNPs of the raccoon dog *SPRN* gene (Table 4). The haplotype present in the highest proportion was CADID (37.2%) followed by CADDD (30.8%) and CAIID (20.3%).

### 3.2. Comparison of the SPRN Gene Polymorphisms Across Various Species

**Figure 2:**
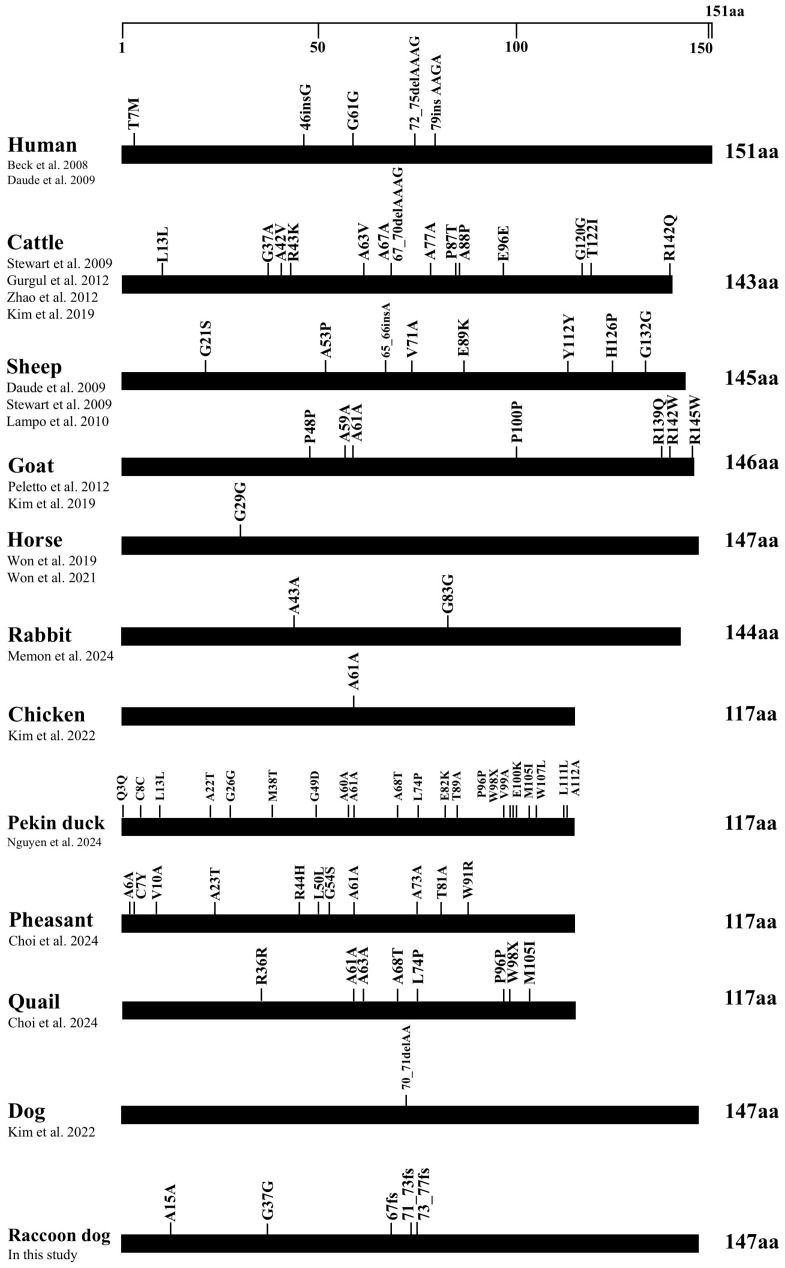
Distribution of genetic polymorphisms in the open reading frame (ORF) of the *SPRN* gene across various species. The figure illustrates the reported genetic polymorphisms of the *SPRN* gene in humans [3,9], cattle [24,25,26,27], sheep [3,24,28], goats [29,30], horses [31,32], rabbits [33], chickens [34], Pekin ducks [35], pheasants [36], quails [37], dogs [38], and raccoon dogs. The outlined horizontal bar represents the length of the amino acid sequence in the *SPRN* gene.

We compared and analyzed the distributions of genetic variations identified in the ORF of the *SPRN* gene in several species. The same amino acid length was found in dogs and raccoon dogs (147 amino acids). Prion disease-resistant species, including horses, rabbits, chickens, and dogs, do not possess non-synonymous SNPs. Similarly, raccoon dogs do not possess non-synonymous SNPs. In raccoon dogs, we identified two synonymous SNPs and three insertion/deletion polymorphisms (Figure 2).

### 3.3. Multiple Sequence Alignments of Sho Across Different Species

**Figure 3:**
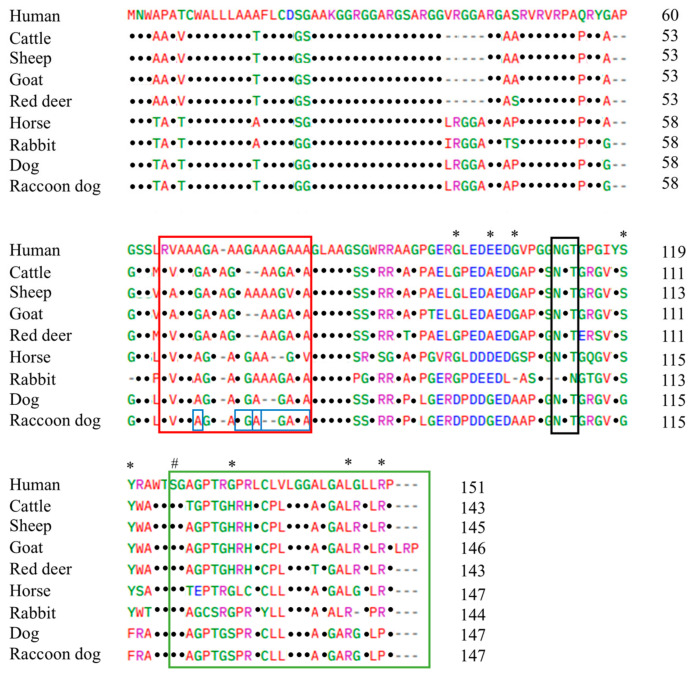
Multiple sequence alignment of Sho in various animals. Colors represent the chemical properties of amino acids: blue for acidic; red for small and hydrophobic; magenta for basic; green for hydroxyl, sulfhydryl, amine and glycine. Dots indicate amino acids that are identical to those in the first row. Asterisks represent canine-specific Sho amino acids. Sharp indicates the omega site of the glycosylphosphatidylinositol (GPI) anchor. The red box highlights the region of interaction between Sho and prion protein (PrP), the black box marks the NXT glycosylation motif, the green box highlights the signal sequence of the GPI anchor, and the blue box highlights genetic variations discovered in this current study.

We conducted multiple sequence alignments of Sho amino acid sequences, including those from humans, cattle, sheep, goats, red deer, horses, rabbits, dogs, and raccoon dogs. Prion disease-resistant animals include horses, rabbits, and dogs. Dogs and raccoon dogs share the same amino acid sequences (Figure 3). Eight canine-specific Sho amino acids (marked with asterisks), including Asp95, Gly99, Ala102, Gly115, Phe116, Ser128, Arg142, and Pro146, were shown in the Sho of both the dog and the raccoon dog (Figure 3). Notably, we observed a high level of conservation across all species in the PrP interaction domain of Sho (shown in red box), the NXT glycosylation motif (shown in black box), and the omega site (serine) as well as the signal sequence (shown in green box) within the C-terminal domain (Figure 3).

### 3.4. Prediction of the 3D Structure of Raccoon Dog Sho Executing the Insertion/Deletion Polymorphism

**Figure 4:**
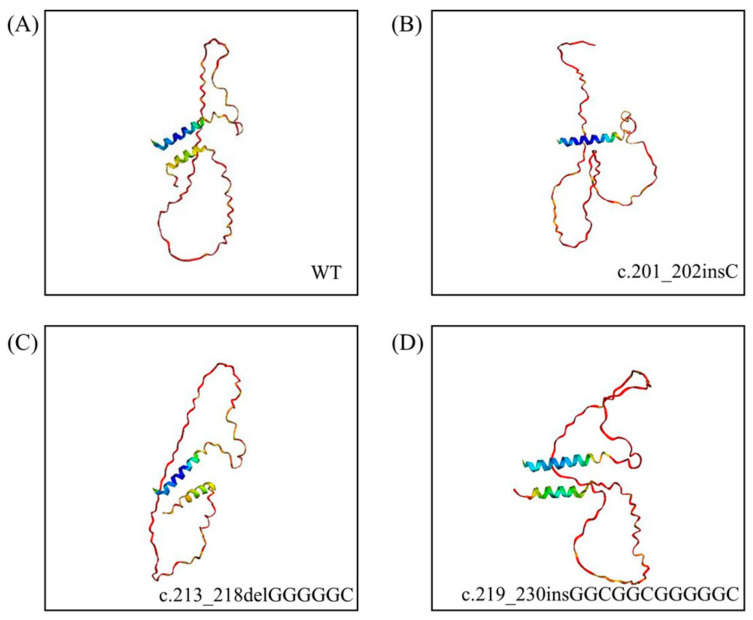
Prediction of the 3D structures of the shadow of prion protein (Sho) in raccoon dogs. (**A**) Predicted 3D structure of wild-type Sho in raccoon dogs. (**B**) Predicted 3D structure of raccoon dog Sho with the c.201_202insC. (**C**) Predicted 3D structure of raccoon dog Sho with the c.213_218delGGGGGC. (**D**) Predicted 3D structure of raccoon dog Sho with the c.219_230insGGCGGCGGGGGC. Red indicates very low confidence, yellow indicates low confidence, green indicates moderate confidence, sky blue indicates high confidence, and blue indicates very high confidence.

AlphaFold2 was utilized to predict the 3D structure of the raccoon dog Sho. The structures of Sho dog and raccoon dog exhibited the same shape as previously reported [38]. In addition, we performed the 3D structure of raccoon dog Sho with the insertion/deletion polymorphisms (Figure 4). Two α-helices were predicted to be connected by a coil in wild-type and insertion/deletion polymorphisms raccoon dog Sho except for the c.201_202insC variant that only had one α-helix.

### 3.5. In Silico Evaluation of the Impact of Polymorphisms in the Raccoon Dog SPRN Gene

To assess the functional impact of insertion/deletion polymorphisms in raccoon dog Sho, we used MutPred-Indel (Table 5). Based on MutPred-Indel analysis, the c.213_218delGGGGGC and c.219_230insGGCGGCGGGGGC polymorphisms received scores of 0.26036 and 0.24008, respectively, suggesting benign effects.

## 4. Discussion

Raccoon dog Sho and dog Sho share the same amino acid sequence [38,39]. To clarify whether prion disease resistance is an unusual characteristic of dogs or a general feature of a Canidae family, we have investigated the *SPRN* gene in raccoon dogs. The *SPRN* gene is essential in influencing susceptibility to prion diseases [9]. The Sho protein is an identified glycoprotein that appears in the neurons of the CNS, and it is broadly suggested that the CNS is the target region of prion disease [40]. Sho protein not only decreases the neurotoxic effects of PrP but also regulates cerebrally expressed prion-like protein (Doppel) in the brains of mice, exhibiting a similar neuroprotective role alongside PrP [10]. In addition, previous studies have indicated that *SPRN* polymorphisms are linked to susceptibility to prion diseases, including CJD, scrapie, and BSE [27]. In our previous study, we investigated the four synonymous SNPs of the raccoon dog *PRNP* gene [20]. Thus, in the current study, we examined the genetic polymorphisms of the *SPRN* gene in raccoon dogs, which are potentially resistant to prion diseases.

In the present study, we found novel SNPs using amplicon sequencing, including two synonymous SNPs within the ORF region of the *SPRN* gene. In addition, we found three insertion/deletion polymorphisms in the ORF of the raccoon dog *SPRN* gene, as shown in Figure 1 and detailed in Table 1. To determine the association between raccoon dog *SPRN* and *PRNP*, we conducted LD analysis using our previous study [20]. It was not expected that there would be strong linkage disequilibrium between *SPRN* and *PRNP* polymorphisms in raccoon dogs. Interestingly, all *SPRN* SNPs showed weak LD with *PRNP* SNPs in horses and pheasants, both prion disease-resistant animals [31,36].

To illustrate how the similarities or differences between raccoon dogs and dogs correspond to resistance mechanisms, we analyzed the distribution of genetic variations and found that both dogs and raccoon dogs share the same amino acid length (147 amino acids) and sequence. Furthermore, the structures analysis showed that the Sho protein in both species displayed identical shapes. In a previous study, we also reported one insertion/deletion polymorphism in the ORF of the dog *SPRN* gene [38]. This polymorphism is located in the region where Sho interacts with PrP in the dogs. The dog Sho protein with the insertion/deletion allele showed a higher binding free energy of Sho and PrP compared to the wild-type dog Sho. This finding suggests that the Sho protein with the insertion/deletion polymorphism forms a less stable complex than the wild-type Sho. Additionally, the three insertion/deletion polymorphisms found in the ORF of the raccoon dog *SPRN* gene are also located in the interaction region of Sho and PrP. These data suggest that raccoon dogs and dogs may reveal the same resistance mechanisms to prion disease. Insertion/deletion variants demonstrated greater efficiency and broader application compared to other molecular markers, including SNPs and microsatellites [41]. Susceptibility to scrapie in goats is related to insertion polymorphisms in the 3′ untranslated region (UTR) of the caprine *SPRN* gene [30]. However, the insertion/deletion polymorphism in the 3′ UTR of the *SPRN* gene has not been investigated in dogs and raccoon dogs. Further studies are needed to determine whether insertion/deletion polymorphisms are associated with other unknown functional polymorphisms in the *SPRN* gene. Moreover, it is essential to analyze the association between polymorphism counts and prion resistance mechanisms.

We also assessed the impact of polymorphisms on raccoon dog Sho using MutPred-Indel. However, this substitution was not predicted to cause notable changes with a benign score. In human genetic diseases, short insertion/deletion variations expanded the range of genetic markers. Several studies have shown that smaller insertion/deletion polymorphisms and common microdeletions may be strongly linked to neuropsychiatric disorders like mental retardation, schizophrenia, and Alzheimer’s disease [42,43,44].

We predicted the 3D structure of Sho using AlphaFold2 and examined the effect of amino acid substitutions on raccoon dog Sho through 3D structure analysis. Prediction of structures with insertion/deletion polymorphisms has two α-helices, except in the case of c.201_202insC Sho. The c.201_202insC Sho variant was predicted to have an altered sequence in the GPI anchor region, which could hinder proper membrane attachment or alter the protein’s interaction dynamics with other cellular components. Protein structure prediction is increasingly recognized as a crucial proteomic tool for elucidating phenomena in modern molecular and cell biology [45], with significant applications in biotechnology and medicine [46]. Thus, the integration of protein structure and biomarkers and genetic screening may prove beneficial in enhancing the precision of diagnosing neurodegenerative diseases such as prion disease. Taken together, raccoon dogs seem to have potential resistance to prion diseases; further studies are required to verify this potential.

## 5. Conclusions

In the present study, two novel synonymous SNPs and three insertion/deletion polymorphisms of the *SPRN* gene in raccoon dogs have been identified. We also evaluated the impact of insertion/deletion polymorphisms using *in silico* tools indicating a benign effect. To our knowledge, this is the first study to explore the genetic polymorphisms and structural characteristics of the *SPRN* gene in raccoon dogs.

## Figures and Tables

**Table 1:** Genotype and allele frequencies of shadow of prion protein (*SPRN*) polymorphisms in raccoon dogs.

Polymorphisms	Genotype Frequencies, *n* (%)			Allele Frequencies, *n* (%)		HWE
	M/M	M/m	m/m	M	m	
c.45C > A	60 (93.75)	2 (3.12)	2 (3.12)	122 (95.31)	6 (4.69)	<0.0001
c.111A > T	62 (96.87)	2 (3.12)	0 (0)	126 (98.44)	2 (1.56)	0.8989
Ins/del Variant 1	50 (78.12)	0 (0)	14 (21.87)	100 (78.12)	28 (21.88)	<0.0001
Ins/del Variant 2	33 (51.56)	19 (29.68)	12 (18.75)	85 (66.40)	43 (33.60)	0.0074
Ins/del Variant 3	61 (95.31)	0 (0)	3 (4.69)	122 (95.31)	6 (4.69)	<0.0001

M/M, Major homozygote; M/m, Heterozygote; m/m, Minor homozygote; M, Major allele; m, Minor allele; HWE, Hardy–Weinberg equilibrium. Ins/del Variant 1: c.201_202insC, Ins/del Variant 2: c.213_218delGGGGGC, Ins/del Variant 3: c.219_230insGGCGGCGGGGGC.

**Table 2:** Linkage disequilibrium (LD) analysis of the *SPRN* polymorphisms of raccoon dogs.

	c.45C > A	c.111A > T	Ins/Del Variant 1	Ins/Del Variant 2	Ins/Del Variant 3
c.45C > A	-				
c.111A > T	0.001	-			
Ins/del Variant 1	0.014	0.007	-		
Ins/del Variant 2	0.0	0.002	0.113	-	
Ins/del Variant 3	0.002	0.001	0.014	0.025	-

Ins/del Variant 1: c.201_202insC, Ins/del Variant 2: c.213_218delGGGGGC, Ins/del Variant 3: c.219_230insGGCGGCGGGGGC.

**Table 3:** Linkage disequilibrium (LD) analysis between SNPs of *PRNP* and *SPRN* gene with r^2^ value in raccoon dogs.

	c.45C > A	c.111A > T	Ins/del Variant 1	Ins/del Variant 2	Ins/del Variant 3
c.108G > T	0.001	0.001	0.005	0.009	0.004
c.198T > C	0.019	0.0	0.013	0.006	0.003
c.261A > T	0.0	0.0	0.002	0.016	0.0
c.264C > T	0.001	0.0	0.004	0.031	0.001

The LD analysis was performed using the single nucleotide polymorphism of the raccoon dog *PRNP* gene. Vertical and horizontal axes represent *PRNP* and *SPRN* polymorphisms, respectively. Ins/del Variant 1: c.201_202insC, Ins/del Variant 2: c.213_218delGGGGGC, Ins/del Variant 3: c.219_230insGGCGGCGGGGGC.

**Table 4:** Haplotype frequency of *SPRN* polymorphisms in raccoon dogs.

Haplotype	c.45C > A	c.111A > T	c.201_202insC	c.213_218delGGGGGC	c.219_230insGGCGGCGGGGGC	Frequency (%)
Ht1	C	A	D	I	D	48 (0.372)
Ht2	C	A	D	D	D	39 (0.308)
Ht3	C	A	I	I	D	26 (0.203)
Ht4	C	A	D	I	I	6 (0.047)
Ht5	A	A	D	I	D	4 (0.034)
Ht6	A	A	D	D	D	2 (0.012)
Ht7	C	T	I	I	D	1 (0.008)
Ht8	C	T	D	D	D	1 (0.008)
Ht9	C	A	I	D	D	1 (0.008)

D and I indicate deletion and insertion, respectively.

**Table 5:** Evaluation using an *in silico* tool regarding the effects of insertion/deletion polymorphisms in raccoon dogs.

Polymorphisms	Score	Prediction
c.201_202insC	NA	NA
c.213_218delGGGGGC	0.26036	Benign
c.219_230insGGCGGCGGGGGC	0.24008	Benign

NA, not available.

## Data Availability

All data generated or analyzed during this study are available from the corresponding author upon reasonable request.
